# Using an in-vivo syngeneic spontaneous metastasis model identifies ID2 as a promoter of breast cancer colonisation in the brain

**DOI:** 10.1186/s13058-018-1093-9

**Published:** 2019-01-14

**Authors:** Magdalena Kijewska, Carmen Viski, Frances Turrell, Amanda Fitzpatrick, Antoinette van Weverwijk, Qiong Gao, Marjan Iravani, Clare M. Isacke

**Affiliations:** 1 The Breast Cancer Now Toby Robins Research Centre, The Institute of Cancer Research, 237 Fulham Road, London, SW3 6JB UK; 2 Present address: Oncology Cell Therapy DPU, GSK, Gunnels Wood Road, Stevenage, SG1 2NY UK; 3 Present address: Division of Tumor Biology & Immunology, The Netherlands Cancer Institute, Plesmanlaan 121, 1066 CX Amsterdam, The Netherlands

**Keywords:** Breast cancer metastasis, Brain metastasis, Spontaneous metastasis, Mouse models, 4T1, ID2, ALDH3A1, Intracranial, Intracardiac

## Abstract

**Background:**

Dissemination of breast cancers to the brain is associated with poor patient outcome and limited therapeutic options. In this study we sought to identify novel regulators of brain metastasis by profiling mouse mammary carcinoma cells spontaneously metastasising from the primary tumour in an immunocompetent syngeneic host.

**Methods:**

4T1 mouse mammary carcinoma sublines derived from primary tumours and spontaneous brain and lung metastases in BALB/c mice were subject to genome-wide expression profiling. Two differentially expressed genes, *Id2* and *Aldh3a1*, were validated in in-vivo models using mouse and human cancer cell lines. Clinical relevance was investigated in datasets of breast cancer patients with regards to distant metastasis-free survival and brain metastasis relapse-free survival. The role of bone morphogenetic protein (BMP)7 in regulating *Id2* expression and promoting cell survival was investigated in two-dimensional and three-dimensional in-vitro assays.

**Results:**

In the spontaneous metastasis model, expression of *Id2* and *Aldh3a1* was significantly higher in 4T1 brain-derived sublines compared with sublines from lung metastases or primary tumour. Downregulation of expression impairs the ability of cells to colonise the brain parenchyma whereas ectopic expression in 4T1 and human MDA-MB-231 cells promotes dissemination to the brain following intracardiac inoculation but has no impact on the efficiency of lung colonisation. Both genes are highly expressed in oestrogen receptor (ER)-negative breast cancers and, within this poor prognosis sub-group, increased expression correlates with reduced distant metastasis-free survival. *ID2* expression also associates with reduced brain metastasis relapse-free survival. Mechanistically, BMP7, which is present at significantly higher levels in brain tissue compared with the lungs, upregulates *ID2* expression and, after BMP7 withdrawal, this elevated expression is retained. Finally, we demonstrate that either ectopic expression of *ID2* or BMP7-induced *ID2* expression protects tumour cells from anoikis.

**Conclusions:**

This study identifies *ID2* as a key regulator of breast cancer metastasis to the brain. Our data support a model in which breast cancer cells that have disseminated to the brain upregulate *ID2* expression in response to astrocyte-secreted BMP7 and this serves to support metastatic expansion. Moreover, elevated *ID2* expression identifies breast cancer patients at increased risk of developing metastatic relapse in the brain.

**Electronic supplementary material:**

The online version of this article (10.1186/s13058-018-1093-9) contains supplementary material, which is available to authorized users.

## Introduction

Metastasis of breast cancer to the brain represents an area of high un-met medical need; 15–30% of patients with metastatic breast cancer will develop brain metastases, and the brain is the first site of metastasis in 7–16% of metastatic patients [1]. Survival upon a brain metastasis diagnosis is 4 months to 2 years, with worse prognosis for patients with triple negative (TN) breast cancer or multiple brain lesions, and better prognosis for those with oestrogen receptor-positive (ER^+^) or human epidermal growth factor receptor 2-positive (HER2^+^) disease or a single brain metastasis [2, 3]. The incidence of brain metastasis for breast and other cancers has been increasing, likely due to improved imaging and other diagnostic technologies and more effective systemic and targeted therapies which prolong patient survival by controlling extra-cranial disease. However, agents that control systemic disease often have poor brain penetrance resulting in failure to control disease progression [4, 5].

Over the past decade there has been significant progress in understanding the mechanisms underpinning brain metastasis, including the identification of key molecular events required for successful tumour cell transmigration across the blood-brain barrier and for avoiding apoptosis once the cells have reached the brain parenchyma [1, 5–8]. However, these studies have relied heavily on directly inoculating tumour cells into the left ventricle of the heart and on the use of human cell lines in immunocompromised mice. Such a scenario fails to recapitulate the clinical setting where low numbers of circulating tumour cells will encounter different organs and have to avoid immune attack [7]. To better mimic human disease, we used a model of spontaneous metastasis of 4T1 mouse mammary carcinoma cells in syngeneic, immunocompetent BALB/c mice. We have profiled 4T1 sublines derived from primary tumours and from tumour cells that had disseminated to the lungs and brain and performed in-vivo validation experiments and assessment of clinical datasets. Using this approach, we identified a role for *ID2* and *ALDH3A1* in promoting metastatic colonisation and for *ID2* in promoting brain-specific metastasis.

## Methods

### Cells and reagents

4T1 cells were obtained from the American Type Culture Collection (ATCC), tagged with luciferase using lentiviral particles expressing Firefly luciferase (Amsbio), and grown in Dulbecco’s modified Eagle’s medium (DMEM) supplemented with 10% fetal bovine serum (FBS). MDA-MB-231-Luc cells were obtained from Sibtech and grown in DMEM supplemented with 10% FBS. Where indicated, 4T1-Luc cells were transduced with lentiviral particles expressing H2B-mRFP as previously described [9] and RFP^+^ cells enriched by fluorescence-activated cell sorting (FACS). Cells were short tandem repeats (STR) tested regularly using the StemElite ID system (Promega). Both cell types were routinely tested for mycoplasma and used within 10 passages after resuscitation. Mouse astrocytes were purchased from ScienCell and maintained in astrocyte basal medium supplemented with FBS and astrocyte growth supplement. Recombinant human transforming growth factor (TGF)β-1 and bone morphogenetic protein (BMP)7 were purchased from R&D systems. Details of short hairpin RNA (shRNA) lentiviruses, full length open reading frame (ORF) clone expression systems, quantitative reverse-transcription polymerase chain reaction (RT-qPCR) reagents, and antibodies used in this study are provided in Additional file 1 (Tables S1–S4).

For shRNA knockdown of *Id2 or Aldh3a1*, 5 × 10^4^ 4T1-Luc cells were transduced with lentiviral particles (Sigma; Mission transduction particles) at a multiplicity of infection (MOI) of 2. At 24 h post-transduction, the medium was replaced with fresh medium containing 10% FBS. Stably transduced cells were selected in 2.5 μg/mL puromycin for two passages.

For ectopic expression of *Id2 or Aldh3a1*, 8 μg of bicistronic mammalian expression vector pReceiver-Lv166 mCherry vector with or without full length ORF for mouse *Id2* (EX-Mm03201-Lv166) or *Aldh3a1* (EX-Mm28326-Lv166-GS) purified plasmid, 4 μg of packaging plasmid psPAX2, and 1.5 μg envelope plasmid pMD2.G were co-transfected into the HEK293T cells using OptiMEM and Lipofectamine 2000. At 48 h post-transfections, virus-containing medium was collected and used to directly infect 4T1-Luc or MDA-MB-231-Luc cells. At 72 h post-infection, cells were FACS sorted to enrich for mCherry-positive cells.

### In-vivo experiments

All animals were monitored on a daily basis by staff from the ICR Biological Service Unit for signs of ill health.

To isolate tumour cells disseminated to metastatic sites for gene expression profiling, 1 × 10^4^ 4T1-Luc cells in 50 μL phosphate-buffered saline (PBS) were inoculated subcutaneously into 6- to 8-week-old female BALB/c mice. Once primary tumours reached the maximum (mean diameter ≥ 15 mm) allowable size, the mice were sacrificed. Primary tumours, lungs, and brains were harvested at necropsy. Primary tumours were individually cut into small pieces, homogenized using a McIlwain Tissue Chopper (Campden Instruments), and digested in L-15 medium containing 3 mg/mL collagenase type I at 37 °C for 1 h, followed by digestion with 0.025 mg/mL DNase1 at 37 °C for 5 min. After erythrocyte lysis using Red Blood Cell Lysing Buffer (Sigma), the cell suspension was plated into a 10-cm dish in 10 mL DMEM plus 10% FBS. Individual lungs and brains were placed in 1 mL PBS on a 40-μm sieve in a 6-cm plate, mechanically dissociated by pushing through the sieve, and cultured in 2 mL DMEM plus 10% FBS in 6-cm dishes. When primary tumour-, brain- and lung-derived 4T1 colonies were visible, cells were passaged 3–4 times before RNA was extracted from individual sublines for gene expression profiling.

For experimental metastasis assays, 6- to 8-week-old female BALB/c or NOD SCID gamma (NSG) mice were inoculated with 4T1-Luc or MDA-MB-231 cells. For intracranial inoculations, mice were anaesthetised with isoflurane and injected with 1 × 10^4^ 4T1-Luc cells in 5 μL PBS into the brain at a rate of 2.5 μL tumour cells/min using a stereotaxic frame with pre-defined co-ordinates relative to bregma (*x* = −2 mm, *y* = 1 mm, *z* = −2 mm). At post-mortem, brains were in-vivo imaging system (IVIS) imaged ex-vivo, fixed in 4% paraformaldehyde for 24 h, and paraffin embedded. For intracardiac inoculation, mice were anaesthetized with isoflurane and 5 × 10^4^ 4T1 (BALB/c mice) or 3 × 10^5^ MDA-MB-231 cells (NSG mice) were injected into the left ventricle of the heart in 100 μL PBS. At the end of the experiment, post-mortem tissues were IVIS imaged ex-vivo, fixed in 4% paraformaldehyde for 24 h, and either paraffin embedded or frozen.

For RNA expression analysis of freshly isolated cells, 4T1-Luc-RFP cells were inoculated either subcutaneously (5 × 10^5^ cells), intravenously via the lateral tail vein (1 × 10^5^ cells) or, as described above, intracranially (1 × 10^4^ cells). Then, 9–13 days later, primary tumours, lungs, and brains were collected. Primary tumours were dissociated using the MACS mouse tumour dissociation kit (Miltenyi Biotec), and lungs and brain were dissociated using the MACS lung dissociation kit. RFP-positive 4T1-Luc cells were FACS sorted directly into RLT lysis buffer (Qiagen) for RNA extraction.

For fluorescent imaging of brain sections, whole 4% paraformaldehyde-fixed brains were submerged in 30% sucrose in PBS at 4 °C before moulding in OCT and freezing in dry ice plus isopentane. The frozen brain was cryostat sectioned at 20-μm intervals. For imaging of mCherry-positive cells, sections were defrosted, washed in PBS, DAPI stained, mounted, and scanned using the Vectra 3.0 automated quantitative pathology imaging system (Perkin Elmer).

For histological and immunohistochemical analysis, formalin-fixed paraffin-embedded (FFPE) brain sections were haematoxylin and eosin (H&E) or antibody stained and scanned on the NanoZoomer digital slide scanner (Hamamatsu). Tumour burden was quantified using ImageJ in a coronal section taken at the median level through each brain.

### Gene expression profiling

RNA extracted (RNeasy Mini kit) from independently isolated 4T1 sublines derived from primary tumour (T, *n* = 3), brain metastases (B, *n* = 4), and lung metastases (L, *n* = 3) was subjected to microarray analysis on Mouse WG-6 v2.0 expression BeadChips (Illumina, San Diego, CA, USA). RNA amplification, labelling, and hybridization were performed according to the manufacturer’s instructions at Cambridge Genomic Services. The raw data were extracted using GenomeStudio Software and was processed in R using the lumi package (http://www.bioconductor.org). In brief, data were: 1) filtered to remove any non-expressed probes (detection *p* > 0.01) across samples involved in a given group comparison; 2) transformed using the variance-stabilising transformation; and 3) normalised using the robust spline normalisation method.

Sample relations were estimated using unsupervised hierarchical clustering (Euclidean distance, average linkage) based on 17,550 probes. Two-sample *t* tests (with random variance model) were used to identify differentially expressed genes between 1) L and T, 2) B and L, and 3) B and T sublines using the BRB-Array Tools (https://brb.nci.nih.gov/BRB-ArrayTools) with a threshold of parametric *p* value < 0.001. When multiple probes were mapped to the same gene, the most variable probe measured by interquartile range (IQR) across the samples was selected to represent the gene. Gene expression data are deposited at GEO with the accession number GSE110101.

### RT-qPCR

RNA from cultured cells or whole mouse tissue or from freshly isolated tumour cells was extracted using the RNeasy Mini kit or the RNeasy Plus Micro kit, respectively, according to the manufacturer’s instructions. RNA was eluted in 10–30 μL nuclease-free water. The RNA concentration was measured in a 1-μL sample using the Qubit2.0 Fluorometer (Invitrogen) or the ND-1000 Spectrophotometer (Nanodrop). cDNA was produced by reverse transcribing 150–500 ng RNA using the QuantiTect reverse transcription kit (Qiagen) or SuperScript IV First-Strand Synthesis System (Invitrogen) according to the manufacturer’s instruction. qPCR was performed on 11.25 ng cDNA (4.5 μL) with 0.5 μL Taqman Gene Expression Assay probe and 5 μL 2× qPCR Master mix per well. Relative quantification was performed using QuantStudio Real-time PCR software or on an ABI Prism 7900HT sequence detection system. Each reaction was performed in triplicate. Data were analysed using QuantStudio Real-time PCR or SDS 2.2.1 software, and relative expression levels were normalised, unless otherwise stated, to *B2m/B2M* or *Gapdh* endogenous control, with a confidence interval of 95% for all assays.

### Cell based assays

For spheroid growth assays, 7.5 × 10^2^ cells/well were sorted into U-bottom low adherence 96-well plates (Corning) in DMEM containing 2% FBS. At 7 days post-seeding, the viability of the cells in the three-dimensional tumour spheroids was assessed using CellTiter-Glo (Promega) with luminescence quantified using a Victor X5 plate reader.

For the anoikis assay, 5 × 10^4^ cells/well were seeded into low-adherence six-well plates (Costar) in DMEM containing 2% FBS. At 24 h post-seeding, cells were stained with Annexin V-APC/PI Apoptosis Detection Kit (eBioscience) and analysed using a BD Biosciences LSRII flow cytometer with FACSDIVA and FlowJo software. Cell viability was measured as a proportion of healthy (Annexin-negative, PI-negative) cells.

### Human and mouse datasets

The expression levels of *ID2* and *ALDH3A1* and their relation to the receptor status of ER, progesterone receptor (PR), and HER2 were assessed for breast cancer samples in The Cancer Genome Atlas (TCGA) [10]. The expression level of *BMP7* in non-tumour-bearing mice was assessed in 1) brain astrocytes, neurons, and microglia using the Srinivasan et al. RNAseq dataset [11], and 2) brain microglia and astrocytes using the Kamphuis et al. microarray dataset [12]. Clinical significance (distant metastasis-free survival) of *ID2* and *ALDH3A1* expression in ER^–^ breast cancers was assessed using publicly available data from Gyorffy et al. [13]. Associations of *ID2* and *ALDH3A1* mRNA levels and brain metastasis were tested in four breast cancer datasets (GSE2034, GSE2603, GSE12276, and GSE14020), normalized by MAS5.0, log_2_ transformed, and batch corrected. The tumour subtype information was published in a previous study [14]. The datasets contained 104 ER^–^ breast cancer patients who either had no metastatic relapse (*n* = 71) or brain-only metastatic relapse (*n* = 33). Brain metastasis relapse-free survival analysis was performed using the upper tertile of gene expression to dichotomise the breast cancers.

To assess the expression of *ID2* in primary breast cancers and breast cancer brain metastases, the datasets described in Schulten et al. (GSE100534) [15] and Harrell et al. (GSE26338) [16] were retrieved. GSE26338 contains data deposited from seven different platforms. Samples run on the GPL5325 platform were enriched for metaplastic breast cancers and were therefore excluded.

### Statistical analysis

Statistical analyses were performed using GraphPad Prism 7. Unless stated otherwise, data represent the mean values ± standard error of the mean (SEM). Where the groups followed normal distribution and had equal variances, the significance of the differences of the groups was tested using either unpaired Student’s *t* test (two groups) or one-way analysis of variance (ANOVA; multiple groups) followed by Bonferroni post-hoc testing for correcting multiple comparisons. If groups did not follow a normal distribution, non-parametric Mann-Whitney (two groups) or Kruskal-Wallis (multiple groups) tests were used. Statistical significance was defined as *p < 0.05, **p < 0.01, and ***p < 0.001.

## Results

### Increased *Id2* and *Aldh3a1* expression in breast cancer cells disseminated to the brain

**Fig. 1:**
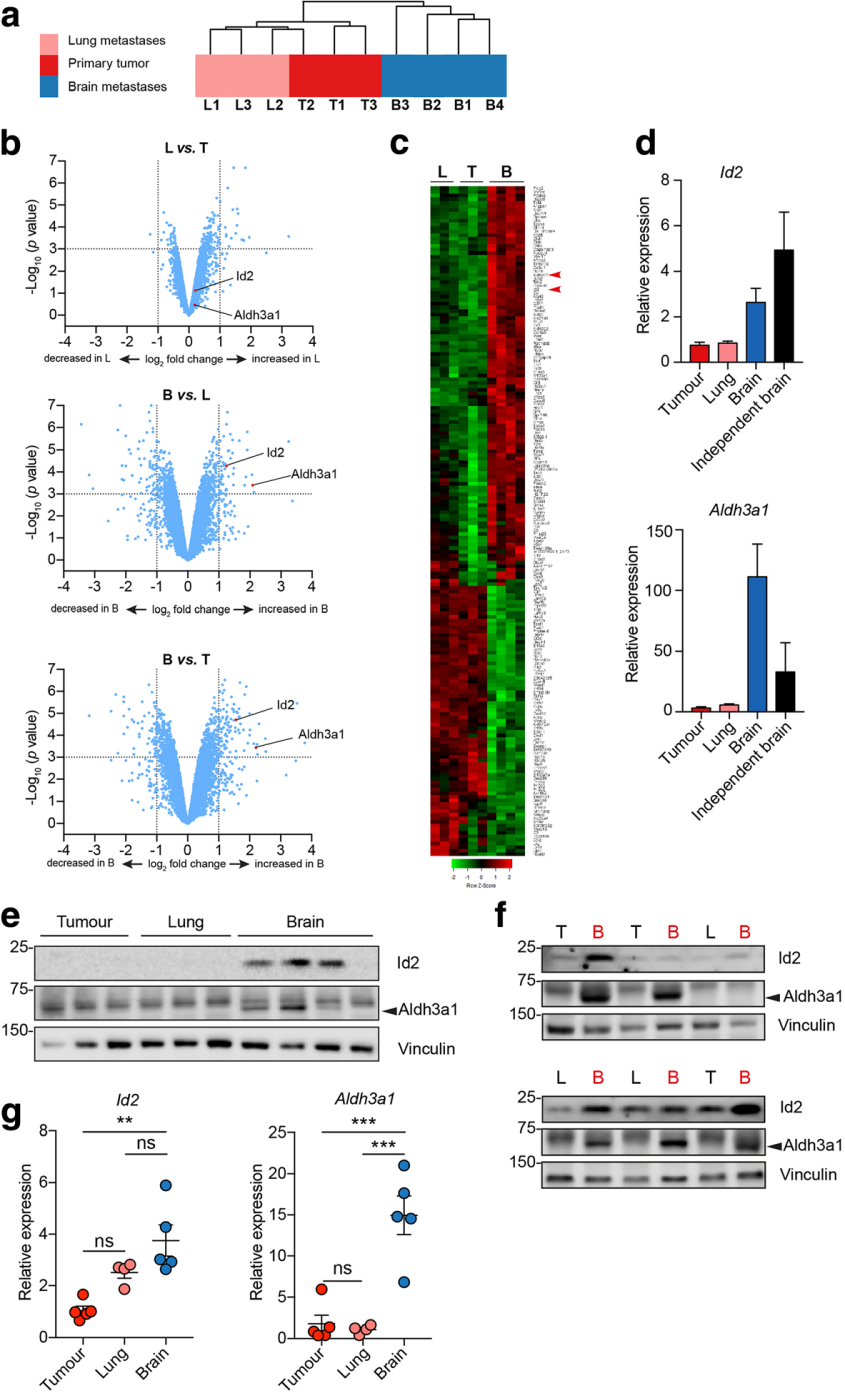
Gene expression profiling of 4T1 sublines from primary and metastatic sites. 4T1-Luc sublines independently isolated from primary tumours (T1–T3), lungs (L1–L3), or brains (B1–B4) were subject to gene expression profiling. **a** Unsupervised hierarchical clustering (Euclidean distance, average linkage) estimating the relation of the independent 4T1 sublines based on 17,550 probes. **b** Volcano plots showing differentially expressed genes (*p* < 0.001) between L vs. T, B vs. L, and B vs. T. *X* axes, gene expression shown on Log_2_^(fold-change)^ scale; *Y*-axes, significance shown on –Log_10_^(*p* value)^ scale. **c** Heat map (Pearson, ward.D2) of 186 genes (with official mouse gene symbol) differentially expressed between B and T, between B and L, or between L and T sublines with an absolute fold change ≥ 2.0, *p* < 0.001. Sublines are in the same order as in Fig. 1a. Arrowheads indicate *Id2* and *Aldh3a1*. See Additional file 1 (Figure S1) for a higher power image including gene names. **d** RT-qPCR analysis of *Id2* and *Aldh3a1* expression in sublines used in a, plus five or six independently isolated brain metastasis sublines. **e,f** Immunoblotting of (**e**) sublines used in a or (**f**) independently isolated 4T1-Luc T, L, and B sublines. Molecular size makers are in kDa. Arrows indicates the lower migrating Aldh3a1 protein. **g** 4T1-Luc-RFP cells were isolated from primary tumours, lungs, and brains of BALB/c mice inoculated subcutaneously, intravenously, or intracranially, respectively. Expression of *Id2* and *Aldh3a1* was analysed by RT-qPCR, *n* = 5 mice per group. Mean ± SEM. **p < 0.01, ***p < 0.001, one-way ANOVA. ns not significant

To establish a model that more closely mimics the clinical scenario, 4T1 mouse mammary carcinoma cells were grown as primary tumours in syngeneic BALB/c mice. At the termination of the experiment, brains, lungs, and primary tumours were individually dissociated to derive 4T1 sublines isolated from different sites. Using this approach, tumour cells were detected in the brains of ~ 25% of the mice, an incidence that is lower than with intracardiac inoculation but which represents a model in which cells have followed the full metastatic cascade. Unsupervised hierarchical clustering of independent 4T1 sublines isolated from primary tumours (*n* = 3), lungs (*n* = 3), and brains (*n* = 4) revealed a separation of the brain-derived sublines from the lung and primary tumour cultures (Fig. 1a). Similarly, two-sample *t* tests of 4T1 sublines (Fig. 1b) identified differentially expressed genes (*p* < 0.001) as follows: 162 genes differentially expressed between lung and primary tumour sublines (122 upregulated in L, 40 downregulated in L), 536 genes differentially expressed between brain and lung sublines (248 upregulated in B, 288 downregulated in B), and 786 genes differentially expressed between brain and primary tumour sublines (379 upregulated in B, 407 downregulated in B). Overall, the tests revealed a closer relationship between the primary tumour and lung sublines than between the brain sublines and either the tumour or lung sublines. A heat map displaying the 186 highly significantly expressed genes (absolute fold change ≥ 2.0, *p* < 0.001) between L and T, between B and L, or between B and T is provided in Fig. 1c, with a higher power image showing all differentially expressed gene names in Additional file 1 (Figure S1).

A shortlist of potential enhancers of brain metastasis was generated by identifying upregulated genes (absolute fold change ≥ 2.0, *p* < 0.001) in B versus T and B versus L, but not between L and T (*n* = 29 genes; Additional file 1: Figure S2). Of these, five genes (*Aldh3a1*, *Bdh2*, *Gpnmb*, *Id2*, and *Uap1l1*) were selected for further validation based on a combination of literature searching and ingenuity pathways analysis. RT-qPCR was performed using RNA isolated from the profiled sublines (B1–B4, L1–L3, T1–T3) plus from five or six independently isolated brain metastasis sublines. *Id2* and *Aldh3a1* were consistently upregulated in the independent brain-derived sublines compared with both the primary tumour and lung-derived sublines (Fig. 1d). Moreover, elevated Id2 and Aldh3a1 protein levels were detected by immunoblotting in the original brain-derived sublines (Fig. 1e) as well as in the independent sublines isolated from the brains of tumour-bearing mice (Fig. 1f).

Finally, to confirm that the expression of *Id2* and *Aldh3a1* is upregulated in tumour cells colonising the brain in-vivo, 4T1 tumour cells were freshly isolated from the brains, lungs, and primary tumours and immediately analysed by RT-qPCR. Consistent with our previous findings (Fig. 1b), both *Id2* and *Aldh3a1* were significantly upregulated in tumour cells isolated from the brain compared with the primary tumour, with a higher expression of these genes in the brain-derived cells compared with those isolated from the lungs (Fig. 1g).

ID2 (inhibitor of differentiation 2 or inhibitor of DNA binding 2) is a helix-loop-helix (HLH)-containing protein that lacks a DNA-binding domain and is one of the four members of the ID family (ID1–ID4). Id proteins dimerise with E protein, Pax, and Ets transcription factors, preventing the formation of DNA-binding transcription complexes [17]. Via their role in inhibiting differentiation and promoting ‘stemness’ and cell proliferation, ID proteins have been implicated in tumour progression in a variety of cancers [18]. ALDH3A1 (aldehyde dehydrogenase 3A1) belongs to the ALDH superfamily consisting of 19 members. In addition to their role in converting both cytotoxic endogenous and exogenous aldehydes to their corresponding carboxylic acids in an NAD(P)^+^- dependent manner [19], ALDH activity is a commonly used marker for identifying cancer cell populations with increased stem or stem-like properties [20, 21]. Although the majority of these studies have focussed on ALDH1A1, ALDH3A1 has been reported to be upregulated in prostate cancer stem cells and in metastatic lesions compared with primary tumours [22] as well as being required for stem cell maintenance and resistance to cytotoxic drugs [23, 24].

### Elevated *Id2* and *Aldh3a1* expression promotes tumour cell colonisation in the brain

**Fig. 2:**
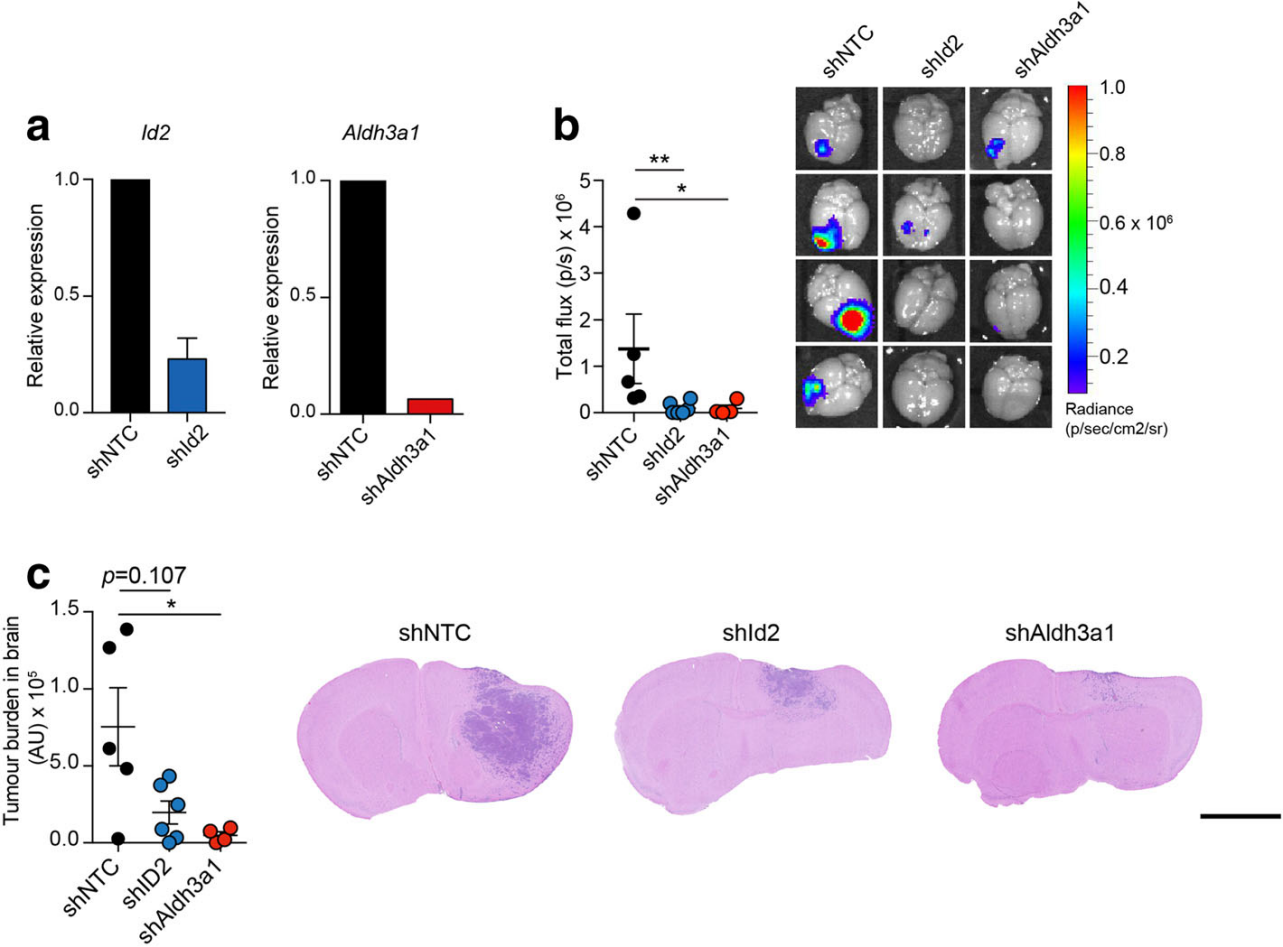
Downregulation of *Id2* or *Aldh3a1* expression impairs tumour growth in the brain. **a** RT-qPCR analysis of *Id2* (left panel) and *Aldh3a1* (right panel) expression in 4T1-Luc cells transduced with lentiviral non-targeting control (shNTC) small hairpin RNA (shRNA) or shRNAs targeting Id2 (shId2) or Aldh3a1 (shAldh3a1), *n* = 3. **b** 1 × 10^4^ 4T1-Luc cells were inoculated intracranially into BALB/c mice, *n* = 4–6 mice per group ± SEM. **p < 0.01, *p < 0.05, Kruskal-Wallis test. At day 9 whole brains were collected at necropsy and subjected to ex-vivo IVIS imaging (left panel), and IVIS images are shown (right panel). **c** Brains from b were fixed, paraffin embedded, and sectioned for histological (H&E) quantification of tumour burden (left panel). Mean ± SEM. *p < 0.05, Kruskal-Wallis test. Right panel shows representative histological sections. Scale bar = 2.5 mm

**Fig. 3:**
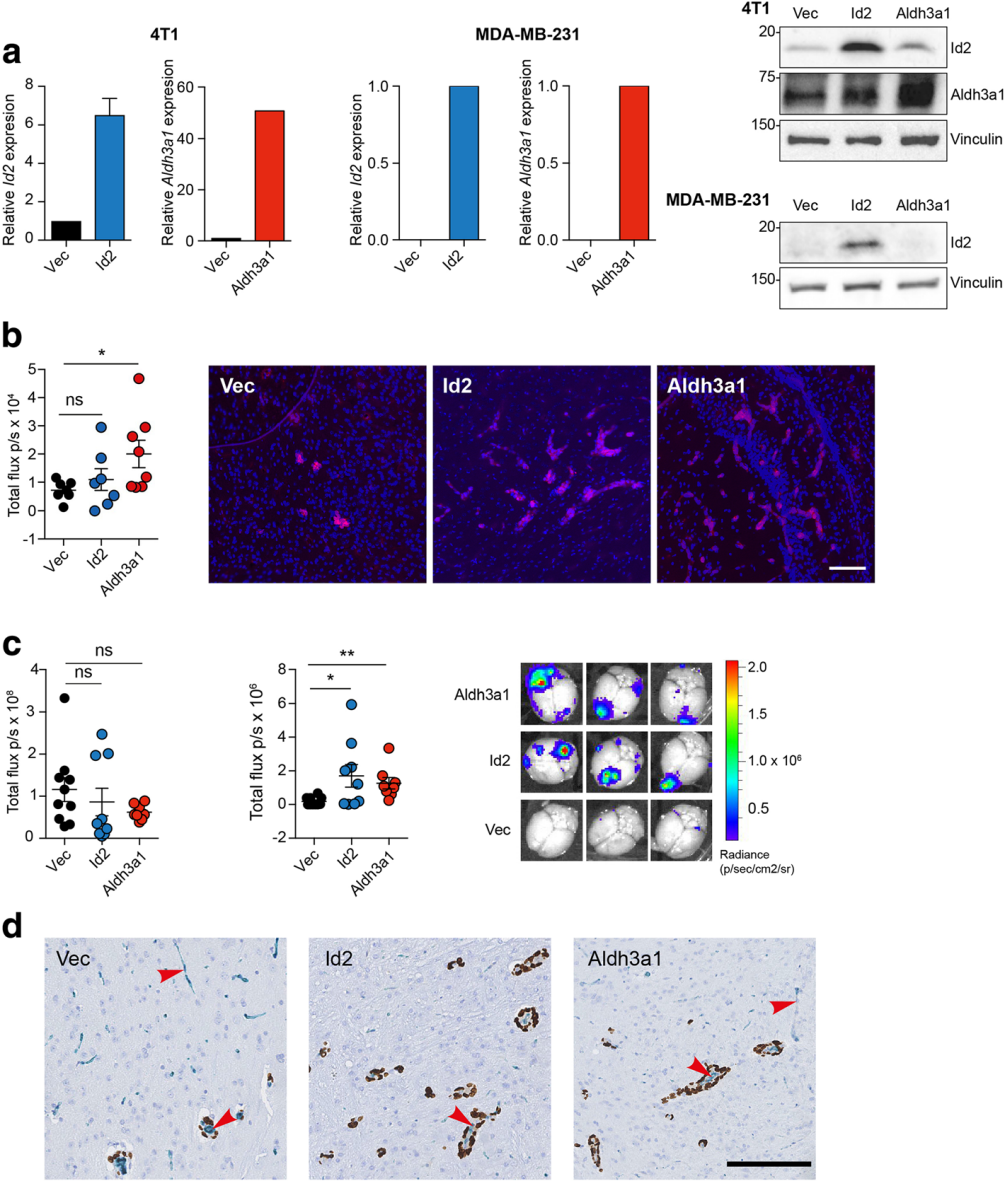
Increased *Id2* or *Aldh3a1* expression enhances metastatic colonisation of the brain following intracardiac inoculation. **a** 4T1-Luc or MDA-MB-231 cells with ectopic expression of *Id2* or *Aldh3a1* or transduced with mCherry vector (Vec) alone. Left panels show RT-qPCR analysis of *Id2* and *Aldh3a1* expression. Right panel shows Id2 and Aldh3a1 protein levels assessed by immunoblotting. **b** BALB/c mice inoculated via the left ventricle with 5 × 10^4^ 4T1-Luc cells, *n* = 7 or 8 mice per group. At day 9, whole brains were collected at necropsy and subject to ex-vivo IVIS imaging. Mean ± SEM. *p < 0.05, Kruskal-Wallis test. Right panel shows representative images of mCherry fluorescent tumour cells in brain sections. Scale bar = 100 μm. **c** 3 × 10^5^ MDA-MB-231-Luc cells were inoculated into the left ventricle of NSG mice, *n* = 9 to 10 mice per group and the experiment terminated on day 23, with organs subject to ex-vivo IVIS imaging. Shown are quantification of ex-vivo IVIS signals in the lungs (left panel, *p* = ns) and brains (middle panel, *p < 0.05, **p < 0.01), Kruskal-Wallis test. Right panel shows representative IVIS images of brains. **d** Sections of fixed and paraffin-embedded brains from c were co-stained for endothelial cell markers endomucin and CD31 (both in blue) and the human nuclear envelope marker lamin A/C (brown). Scale bar = 100 μm. Arrowheads indicate endomucin/CD31 stained vasculature. ns not significant

To assess whether Id2 and/or Aldh3a1 play a role in promoting the growth of tumour cells in the brain, 4T1-Luc cells were transduced with lentiviruses containing non-targeting control shRNA (shNTC) or shRNAs targeting *Id2* (shId2) or *Aldh3a1* (shAldh3a1) (Fig. 2a) and inoculated intracranially into BALB/c mice. Ex-vivo IVIS imaging of the brains post-mortem revealed a significant reduction in tumour burden in both the shId2 and shAldh3a1 groups (Fig. 2b). This finding was confirmed by histological examination of brain sections (Fig. 2c). Although these data indicate that *Id2* and *Aldh3a1* expression is required for efficient tumour growth in the brain, they do not address whether these genes play a role in promoting brain colonisation of tumour cells from the circulation. Due to the lack of mouse models with which to reproducibly monitor spontaneous breast cancer metastasis, we ectopically expressed *Id2* or *Aldh3a1* in mouse 4T1-Luc cells and in the human breast cancer cell line, MDA-MB-231-Luc (Fig. 3a), and performed intracardiac inoculation of the manipulated cells into BALB/c and NSG mice, respectively. For the 4T1-Luc cells, ectopic expression of *Aldh3a1* resulted in a significant increase in tumour cell colonisation of the brain as monitored by ex-vivo IVIS imaging. Increased *Id2* expression also increased brain colonisation but this did not reach significance (Fig. 3b). However, examination of brain sections revealed that, compared with the control cells, cells with either ectopic *Id2* or *Aldh3a1* expression were better spread into the perivascular space of the brain parenchyma (Fig. 3b, right panels). Consistent with the gene expression profiling (Fig. 1), ectopic expression of either *Id2* or *Aldh3a1* had no impact on the level of tumour burden in the lung (Fig. 3c, left panel) but significantly increased tumour burden in the brain (Fig. 3c, middle and right panels). Finally, we examined the distribution of tumour cells in the brain by co-staining sections with the endothelial markers endomucin and CD31, and an antibody against human lamin A/C to detect the MDA-MB-231-Luc cells. As has been previously reported [25–28], the majority of tumour cells had extravasated into the brain parenchyma and were growing in the perivascular space (Fig. 3d).

### Elevated *ID2* expression associates with poor outcome in patients with brain metastases

**Fig. 4:**
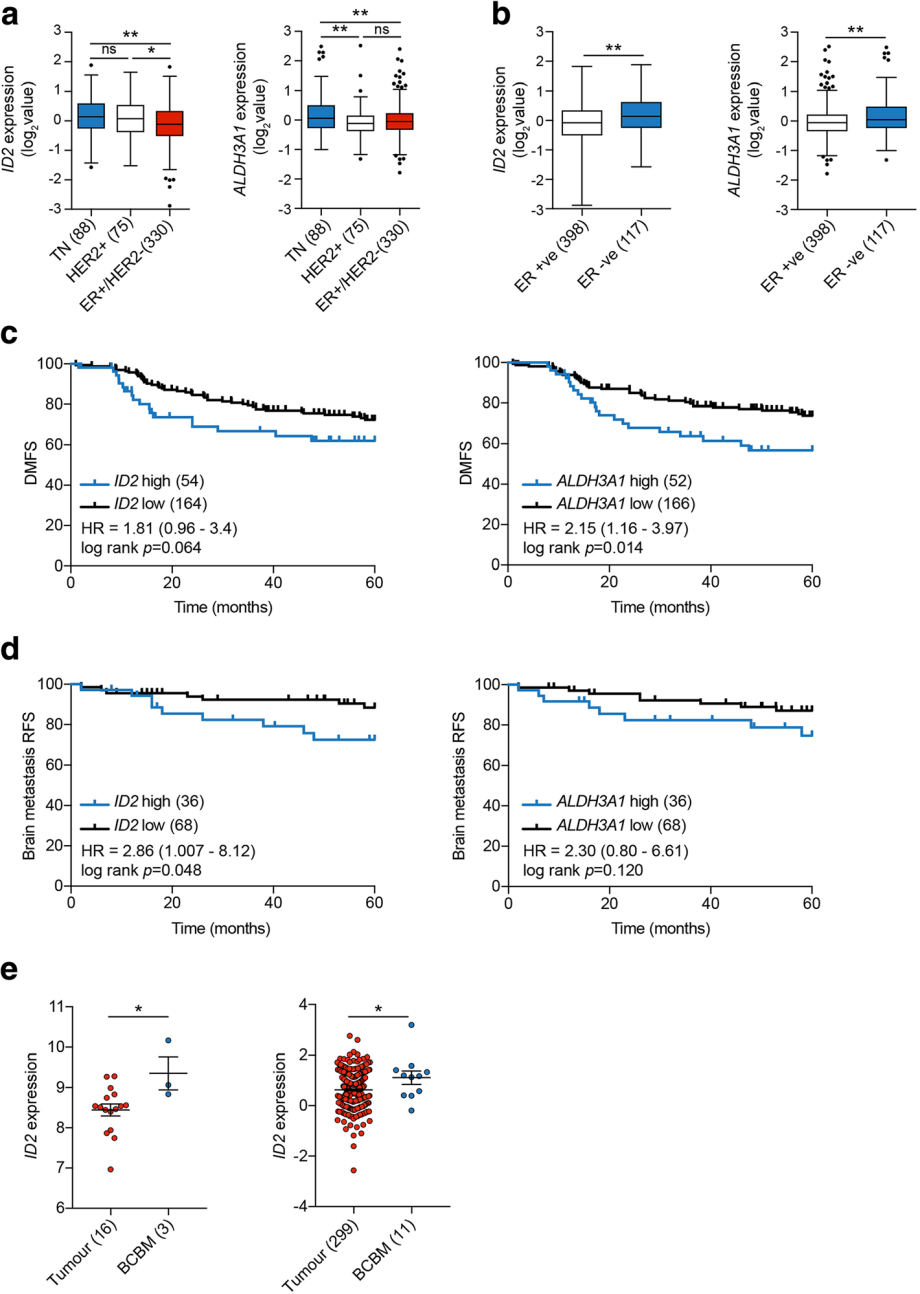
Elevated *ID2* expression correlates with poor patient outcome. **a**,**b** Tukey plots representing the expression of *ID2* and *ALDH3A1* in the TCGA datasets based on receptor status. Box indicates the first and third quartiles, the bar indicates the median, whiskers indicate 1.5 interquartile range (IQR), and dots indicate outliers. The number of samples is shown in parentheses. **a** Adjusted *p* values determined using one-way ANOVA followed by Tukey’s multiple comparisons test. **p < 0.01, *p < 0.05. **b** *p* values determined using Mann-Whitney test (*p* < 0.0016 for *ID2*, *p* < 0.0028 for *ALDH3A1*). **c** Kaplan-Meier analysis of breast cancer-specific distance metastasis-free survival (DMFS) in the 218 oestrogen receptor (ER)-negative tumours of the Gyorffy et al. dataset [13]. The number of samples in each group, hazard ratios (HR), and log rank Mantel-Cox *p* values are shown. **d** Kaplan-Meier analysis of brain metastasis relapse-free survival (RFS) in the cohort of 104 ER-negative tumours from patients who had either no metastatic relapse or brain-only relapse (see Methods). HR and log rank Mantel-Cox *p* values are shown. **e** Comparison of *ID2* expression in primary breast cancers and breast cancer brain metastases (BCBM) in the Schulten et al. [15] (left panel) and Harrell et al. [16] (right panel) datasets. The number of samples is shown in parentheses. Data represent normalised expression values ± SEM. HER2 human epidermal growth factor 2, ns not significant, TN triple-negative

Next, we addressed the clinical relevance of these findings for human breast cancer patients. Examination of the TCGA breast cancer dataset revealed higher levels of *ID2* and *ALDH3A1* expression in triple-negative (ER^–^/PR^–^/HER2^–^) breast cancers compared with HER2^+^ and ER^+^/HER2^–^ breast cancers (Fig. 4a), and in ER^–^ versus ER^+^ breast cancers (Fig. 4b). Moreover, in ER^–^ breast cancers, elevated expression of either *ID2* or *ALDH3A1* was associated with reduced distant metastasis-free survival (Fig. 4c). More importantly, elevated expression of *ID2* was associated with reduced brain metastasis free-survival, whereas there was no significant association of *ALDH3A1* expression levels with outcome in these patients (Fig. 4d). Consistent with our mouse experimental studies demonstrating elevated *Id2* expression in cells isolated from brains compared with primary tumours (Fig. 1g), interrogation of human datasets containing gene expression profiling of primary breast cancer and breast cancer brain metastases revealed a significantly increased level of *ID2* expression in the brain samples (Fig. 4e). As a consequence, we focussed the remainder of our studies on *ID2*.

### BMP7 signalling promotes *Id2* expression and enhances cell survival

**Fig. 5:**
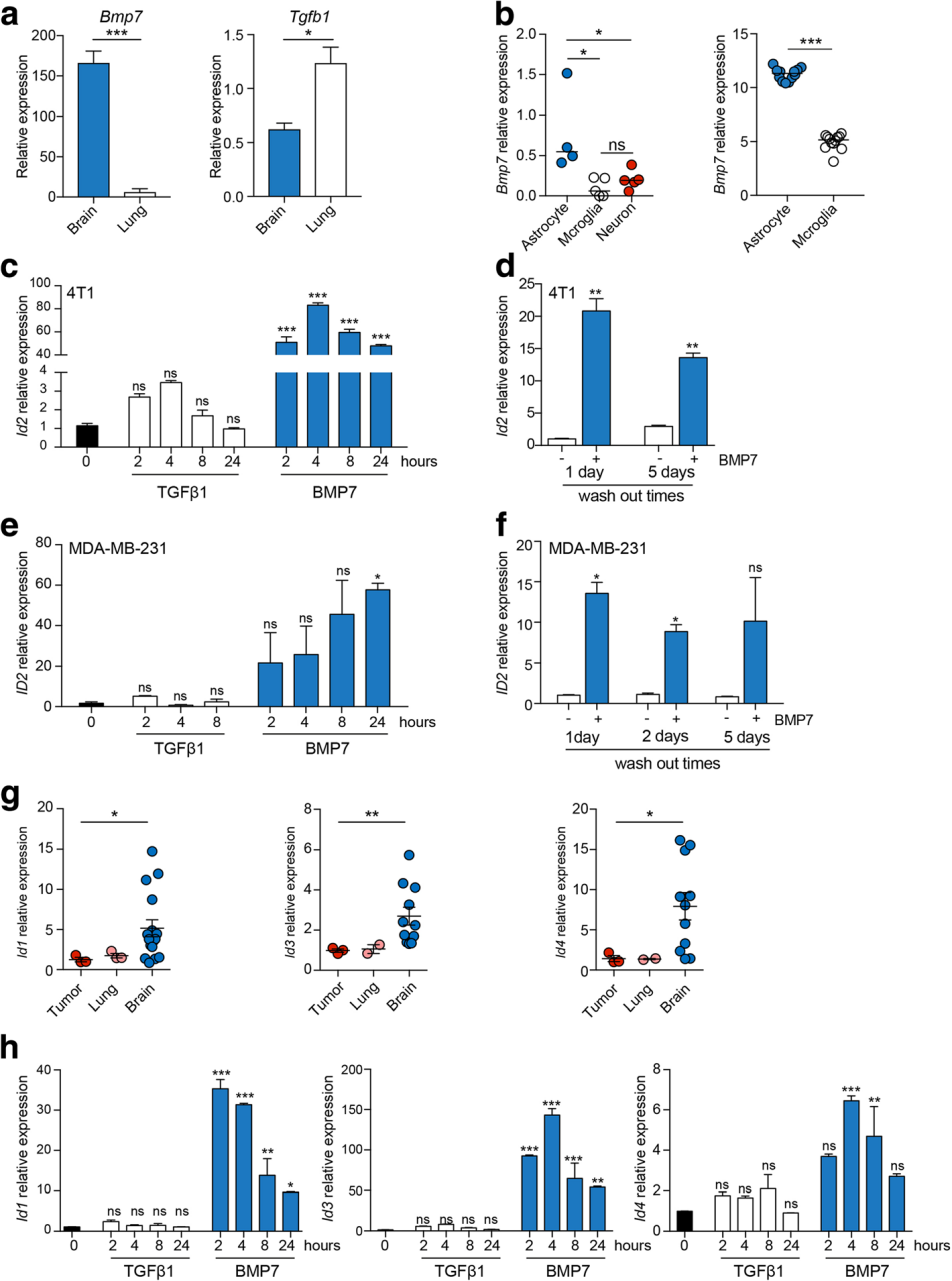
*Id2* expression is induced by bone morphogenetic protein (BMP)7 but not transforming growth factor (TGF)β1. **a** RT-qPCR analysis of *Bmp7* and *Tgfb1* expression in brains and lungs from naive BALB/c mice (*n* = 3 independent samples). ***p < 0.001, *p < 0.05, Student’s *t* test. **b** Expression levels of *Bmp7* in mice cell types isolated from non-tumour-bearing mice in the Srinivasan RNAseq dataset [11] (GSE75246; *n* = 4 or 5 samples per cell type) and Kamphuis dataset [12] (GSE74614; *n* = 12 samples per cell type). Scatter plots represent median. *p < 0.05, ***p < 0.001, Mann Whitney test. **c** RT-qPCR analysis of *Id2* expression in 4T1-Luc cells treated with recombinant TGFβ1 (5 ng/mL) or BMP7 (300 ng/mL) for 2–24 h. Data represent 2 wells/condition, mean ± SEM, and are shown relative to the untreated sample. ***p < 0.001, one-way ANOVA. **d** 4T1-Luc cells were treated with BMP7 (300 ng/mL) for 24 h and cells were washed and incubated in complete media for a further 1 or 5 days before *Id2* expression was assessed by RT-qPCR. Data represent 2 wells/condition, mean ± SEM. **p < 0.01, Student’s *t* test. **e**,**f** *ID2* expression levels in MDA-MB-231-Luc cells treated as described in b and c. *p < 0.05, one-way ANOVA and Student’s *t* test. **g** RT-qPCR analysis of *Id1*, *Id3*, and *Id4* expression in 4T1-Luc tumour sublines independently isolated from primary tumours, lungs, and brains, relative to *Gapdh*. *p* values generated using Mann-Whitney test between tumour and brain. *p < 0.05, **p < 0.01 (lungs were not included in statistical analysis as *n* = 2 samples). **h** RT-qPCR analysis of *Id1*, *Id3*, and *Id4* expression levels in 4T1-Luc cells treated with TGFβ1 (5 ng/mL) or BMP7 (300 ng/mL) for 2–24 h. Data represent 2 samples per time point, mean ± SEM and are shown relative to untreated samples. *p < 0.05, **p < 0.01, ***p < 0.001, one-way ANOVA. ns not significant

It has been reported previously that ID2 expression is positively regulated by BMP7 signalling but negatively regulated by TGFβ1 [29, 30]. Interestingly, in non-tumour-bearing mice, *Bmp7* expression is substantially higher in the brain than the lungs, whereas the expression of *Tgfb1* is higher in the lungs compared with the brain (Fig. 5a). Examination of publicly available datasets revealed a significant enrichment of *Bmp7* expression in brain astrocytes compared with microglia or neurons (Fig. 5b). To directly address whether BMP7 can induce *Id2* expression in the tumour cells, 4T1-Luc cells were untreated or treated for 2 to 24 h with either BMP7 or TGFβ1. BMP7, but not TGFβ1, treatment promoted a profound increase in *Id2* mRNA levels (Fig. 5c). Moreover, this elevated expression persisted for up to 5 days after the removal of BMP7. These effects were not restricted to the mouse 4T1-Luc cells since equivalent results were obtained with the MDA-MB-231 cells (Fig. 5e, f). We also investigated the expression of other Id family members, *Id1*, *Id3*, and *Id4*. All family members showed elevated expression in the 4T1-Luc sublines isolated from the brains of tumour-bearing mice compared with the lungs or primary tumour sublines (Fig. 5g). In addition, expression of *Id1* and *Id3*, and to a lesser extent *Id4*, was dramatically elevated following BMP7, but not TGFβ1, treatment (Fig. 5h).

**Fig. 6:**
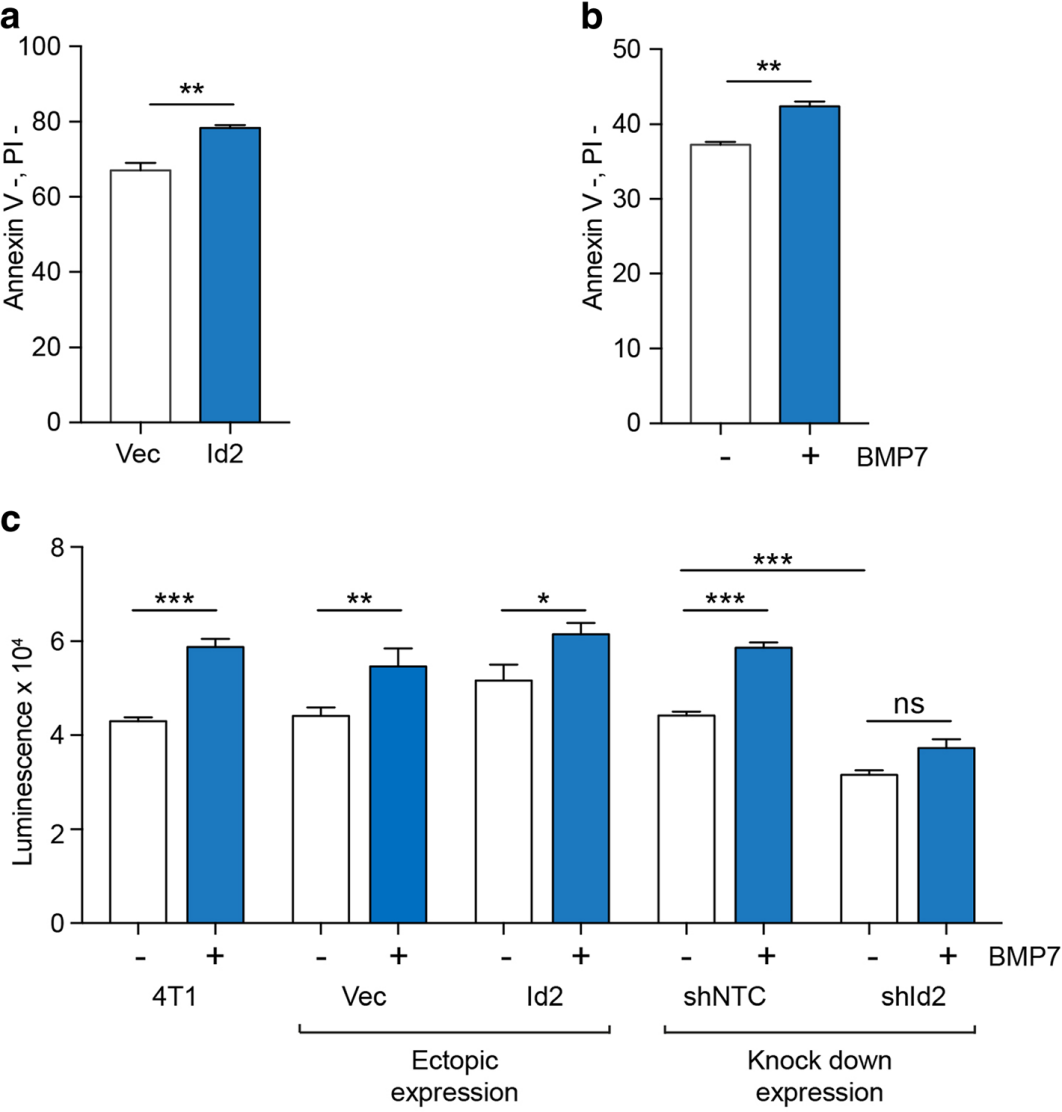
*Id2* expression protects against apoptosis. **a** 5 × 10^4^ 4T1-Luc cells expressing vector alone (Vec) or with ectopic expression of *Id2* were cultured in six-well low-adherence plates in 2% FBS for 24 h before annexin V/PI staining. Data shown as percent of healthy cells (annexin V^–^, PI^–^) remaining, *n* = 3. Mean ± SEM. **p < 0.01, Student’s *t* test. **b** Parental 4T1-Luc cells cultured in suspension as described in a in the presence of absence of bone morphogenetic protein (BMP)7 (300 ng/mL) for 24 h prior to annexin V/PI staining. Data shown as percent of healthy cells (annexin V^–^, PI^–^) remaining, three samples per condition. Mean ± SEM. **p < 0.01, Student’s *t* test. **c** 750 parental 4T1-Luc cells (4T1) or 4T1-Luc cells transduced with empty vector (Vec) or with ectopic expression of *Id2*, or 4T1-Luc cells transduced with non-targeting control short hairpin RNA (shNTC) or shRNA targeting *Id2* (shId2) were sorted into low-adherence 96-well U-bottomed plates in DMEM plus 2% FBS and cultured in the presence or absence of BMP7 (300 ng/mL) for 7 days before viability was monitored by CellTiter-Glo. Data from *n* = 5 wells/sample. *p < 0.05, **p < 0.01, ***p < 0.001, two-way ANOVA with Tukey’s post-test. ns not significant

Finally, we determined the mechanism by which Id2 might promote colonisation of the brain. Tumour cells disseminating to the brain will find themselves in a foreign environment and, in particular, an environment devoid of many of the extracellular matrix components found in primary tumours or in the lungs [31]. To mimic a matrix-poor environment, 4T1-Luc cells were cultured in low-adherence plates. Under these conditions, either ectopic expression of *Id2* (Fig. 6a) or treatment with BMP7 to induce *Id2* expression (Fig. 6b) resulted in a higher proportion of non-apoptotic 4T1-Luc cells remaining after 24 h. To extend these observations, 4T1-Luc cells were cultured as three-dimensional tumour spheroids by plating cells into low-adherence U-bottomed plates (Fig. 6c). Treatment with BMP7 of either 4T1-Luc parental cells, 4T1-Luc cells transduced with empty vector (Vec), or with a non-targeting control shRNA (shNTC) resulted in increased viability of cells within the spheroids. Conversely, knockdown of *Id2* expression (shId2) impaired viability and impaired the ability to respond to BMP7 treatment.

## Discussion

In this study, we generated a set of 4T1 sublines derived from primary tumours and from tumour cells that had spontaneously disseminated to either the brain or lungs of BALB/c mice. By pair-wise gene-expression profiling, we sought to identify differentially expressed transcripts specifically associated with metastasis to the brain. Moreover, as these sublines were derived in the context of a spontaneous metastasis model where the number of circulating tumour cells reaching secondary sites is very limited, we reasoned that differentially expressed genes were more likely to be involved in survival of cells in the brain and the early stages of metastatic colonisation rather than the expansion of established macrometastatic disease.

Two differentially expressed genes were selected for in-vivo validation, *Aldh3a1* and *Id2*. Downregulation of either gene resulted in reduced tumour burden following intracranial inoculation whereas ectopic expression in either mouse or human tumour cells resulted in increased metastatic burden in the brain following intracardiac inoculation but did not promote increased metastasis to the lung.

Tumour cells colonising the brain face unique challenges. First, the tumour cells have to navigate across the blood-brain barrier that separates the brain from the general circulation [32]. Second, the cellular and non-cellular composition of the brain is distinct from that found both in the primary tumour and at other metastatic sites. For example, tumour cells metastasising to the brain have to avoid apoptosis mediated by astrocyte-secreted Fas ligand [26, 33] and detection and elimination by reactive microglia [6]. Similarly, the absence of stromal fibroblasts and fibrillar collagen [31] means that infiltrating tumour cells have to adapt to the foreign extracellular matrix composition. Finally, successful metastasising cells have to adapt to an altered metabolic environment [7, 34]. The brain consumes ~ 20% of the body’s glucose-derived energy but, when blood glucose is low, the brain can adapt its metabolism to use acetate, ketone bodies, or fatty acids as alternative fuels. Metastasising tumours cells need to have metabolic flexibility to survive in this environment.

As has been reported in other studies [25–28], in the models used here it was observed that tumour cells that disseminated to the brain parenchyma remained closely located to the brain capillaries (see Fig. 3). It is now well established that cancer stem cells reside in niche microenvironments, including perivascular niches in the brain [35], and that these niches serve to maintain the tumour cell ‘stemness’ [36]. As both ID2 and ALDH3A1 have been implicated in promoting stem cell-like features in tumour cells [18, 24, 37], their elevated expression may provide an advantage in the early stage of metastatic colonisation by retaining cells in the niche until they are ready to face the full challenges of the brain microenvironment. In addition, among the ALDH family, ALDH3A1 has a specific role in peroxidic aldehyde metabolism [24, 38]. In metabolically challenging conditions, particularly in conditions of high oxidative stress, production of reactive oxygen species (ROS) leads to peroxidation of lipids, which in turn gives rise to cytotoxic lipid aldehydes. The ability of ALDH3A1 to function as lipid aldehyde scavenger [39] would provide a survival advantage for disseminating tumour cells encountering adverse conditions. However, it was notable in this study that, when expression of *ALDH3A1* was examined in breast cancer clinical datasets, high expression was significantly associated with an increased risk of distant metastatic relapse but not with brain-specific relapse (Fig. 4c, d). Consequently, although the models used in this study identified increased *Aldh3a1* expression to be associated with increased brain but not lung metastasis, the clinical data point to a more general pro-metastatic function. In contrast, *ID2* expression in clinical samples correlated with increased distant metastatic relapse and brain-specific metastatic relapse.

ID proteins are negative regulators of basic HLH (bHLH) transcription factors and, additionally, ID2 can bind and over-ride the tumour suppressor function of retinoblastoma (RB) tumour suppressor. In normal cells, these activities underpin the role of ID proteins in inhibiting cell differentiation and promoting cell proliferation. In cancers, ID proteins are found at a higher level than in normal adult tissues where they function to sustain self-renewal of stem or stem-like cancer cells and inhibit apoptosis and entry of tumour cells into senescence [17, 18]. For ID2 it has been shown that expression is required to maintain glioma [40], glioblastoma [41], head and neck [42], and colorectal cancer stem/stem-like cells. Previously it has been demonstrated in normal mouse mammary cells and other non-transformed epithelial cell types that exposure to TGFβ resulted in decreased *ID2* mRNA and protein levels, whereas BMP7 could both over-ride the TGFβ-mediated *ID2* repression and, on its own, promote increased *ID2* expression [29, 30, 43]. BMPs are members of the TGFβ superfamily but, whereas TGFβs bind to the TGFβ type 1 receptors ALK1 and ALK5, BMPs bind to BMP-specific ALK receptors, with BMP7 binding to ALK2 [44].

Given the role reported here for ID2 in promoting breast cancer metastasis to the brain, it was striking that, although levels of *Tgfb1* mRNA were approximately two-fold higher in the normal mouse lung compared with the brain, levels of *Bmp7* mRNA were > 100-fold higher in the brain compared with the lung. Moreover, whereas treatment with TGFβ1 had little impact on *ID2* expression in either mouse mammary carcinoma or human breast cancer cells, BMP7 treatment resulted in a > 50-fold increase in *ID2* mRNA levels and this level of expression was sustained for at least 5 days following BMP7 withdrawal. Together with the demonstration that increased *Id2* expression protects tumour cells from loss of attachment-induced anoikis and promotes growth of three-dimensional tumour spheroids, the data presented here support a model in which spontaneously metastasising breast cancers cells with higher *ID2* expression will have a survival advantage when they disseminate to the brain and that, once in the brain, cells that are receptive to BMP7 signalling to maintain high-level *ID2* expression will be better able to progress to macrometastatic disease by tolerating the harsh pro-apoptotic brain microenvironment.

## Conclusions

The incidence of brain metastasis in breast and other cancers is increasing, yet patient prognosis after a diagnosis of metastatic relapse in the brain remains dismal. This situation is further compounded by the lack of prospective clinical trial data to assess the efficacy of current system therapies in patients with brain metastases [1]. In addition, there is an urgent need to develop methodologies for the identification of patients at high risk of developing brain metastasis and to better understand the unique biology associated with tumour cell colonisation of the brain to identify potential new therapeutic strategies. Using a model that more faithfully recapitulates the dissemination of tumour cells to secondary sites, combined with in-vivo and in-silico validation studies, we identified ID2 as a promising brain metastasis promoter. Future studies will be required to assess whether monitoring ID2 mRNA or protein levels in primary tumours or in plasma samples could provide a prognostic biomarker for patients at higher risk of relapse in the brain. Finally, it is well documented that ID family proteins play a key role in developmental processes and that expression is downregulated in most normal adult tissues but can be re-activated in cancer cells [17, 18]. Although this pattern of expression makes ID proteins potential therapeutic targets, helix-loop-helix proteins are typically described as ‘undruggable’. However, in recent years there have been rapid technological advances in developing inhibitors of protein-protein interaction as well as approaches for targeted protein degradation such as proteolysis targeting chimaeras (PROTACs), bifunctional molecules for hijacking E3 ligases or small molecules that redirect E3 ligase activity [45]. The development of reagents that can inhibit ID2 dimerisation or promote its degradation will, in the future, allow robust assessment of ID2 as a potential therapeutic target to prevent or limit the development of breast cancer brain metastasis.

## Additional file

Additional file 1: **Table S1.** MISSION® shRNA pLKO-puro transduction particles. **Table S2.** Open Reading Frame Clone Expression Systems (Genecopoeia). **Table S3.** Taqman RT-qPCR gene expression assays. **Table S4.** Antibodies used for immunoblotting (IB) and immunohistochemistry (IHC). **Figure S1.** Higher power image of heat map of differentially expressed genes from Fig. 1c. **Figure S2.** Heat map of shortlisted genes. (PDF 1540 kb)

## Abbreviations

ATCC: American Type Culture Collection
B2M: Beta-2-microglobin
BMP: Bone morphogenetic protein
ER: Oestrogen receptor
FACS: Fluorescence-activated cell sorting
FBS: Fetal bovine serum
FFPE: Formalin-fixed paraffin-embedded
GAPDH: Glyceraldehyde-3-phosphate dehydrogenase
H&E: Haematoxylin and eosin
H2B-mRFP: Histone H2B-monomeric red fluorescent protein
HER2: Human epidermal growth factor receptor 2
IVIS: In-vivo imaging system
MOI: Multiplicity of infection
NSG: NOD SCID gamma
ORF: Open reading frame
PR: Progesterone receptor
RT-qPCR: Quantitative reverse-transcription polymerase chain reaction
shRNA: Short hairpin RNA
STR: Short tandem repeats
TCGA: The Cancer Genome Atlas
TGF: Transforming growth factor
