# Supplementary material for: Using an in-vivo syngeneic spontaneous metastasis model identifies ID2 as a promoter of breast cancer colonisation in the brain
Source: Breast Cancer Res. 2019 Jan 14;21:4. doi: 10.1186/s13058-018-1093-9 (PMC6332688; doi:10.1186/s13058-018-1093-9)
Supplement: Supplementary file 1 — Table S1. MISSION® shRNA pLKO-puro transduction particles. Table S2. Open Reading Frame Clone Expression Systems (Genecopoeia). Table S3. Taqman RT-qPCR gene expression assays. Table S4. Antibodies used for immunoblotting (IB) and immunohistochemistry (IHC). Figure S1. Higher power image of heat map of differentially expressed genes from Fig. 1c. Figure S2. Heat map of shortlisted genes. (PDF 1540 kb) [file 13058_2018_1093_MOESM1_ESM.pdf]

Using an *in vivo* syngeneic spontaneous metastasis model identifies ID2 as a promoter of breast cancer colonisation in the brain

Magdalena Kijewska, Carmen Viski, Frances Turrell, Amanda Fitzpatrick, Antoinette van Weverwijk, Qiong Gao, Marjan Iravani and Clare M. Isacke

Additional file 1; Supplementary Tables

Table S1. MISSION® shRNA pLKO-puro transduction particles

Table S2. ORF Clone Expression Systems (Genecopoeia)

Table S3. Taqman RT-qPCR gene expression assays

Table S4. Antibodies for immunoblotting (IB) and immunohistochemistry (IHC)

Additional file 1; Supplementary Figure

Figure S1. Higher power image of Figure 1c

Figure S2. Heat map of shortlisted genes

Table S1 MISSION shRNA pLKO-puro transduction particles

Clone I.D.	Gene targeted	Species targeted	Named
SHC202V	None	None	shNTC
TRCN0000054390	*Id2*	Mm	shId2
TRCN0000042078	*Aldh3a1*	Mm	shAldh3a1

Table S2 ORF clone expression systems

Vector	I.D.	Tag	Species	Gene	Named
pReceiver-LV166	N/A	IRES2-mcherry	N/A		Vec
pReceiver-LV166	Mm03201	IRES2-mcherry	Mouse	Id2 (NM_010496.3)	Id2 OE
pReceiver-LV166	Mm28326	IRES2-mcherry	Mouse	Aldh3a1 (NM_001112725.1)	Aldh3a1 OE

Table S3 Taqman RT-qPCR gene expression assays

Gene symbol	Gene name	Probe set I.D.	Species
B2m	β-2 microglobulin	Mm00437762_m1	Mm
B2M	β-2 microglobulin	Hs99999907_m1	Hs
Gapdh	Glyceraldehyde-3-phosphate dehydrogenase	4352339E	Mm
Id2	inhibitor of DNA binding 2	Mm00711781_m1	Mm
ID2	inhibitor of DNA binding 2	Hs04187239_m1	Hs
Aldh3a1	aldehyde dehydrogenase 3 family member A1	Mm00839312_m1	Mm
ALDH3A1	aldehyde dehydrogenase 3 family member A1	Hs00964880_m1	Hs
Id1	Inhibitor of DNA binding 1	Mm00775963_g1	Mm
Id3	Inhibitor of DNA binding 3	Mm00492575_m1	Mm
Id4	Inhibitor of DNA binding 4	Mm00499701_m1	Mm

Table S4 Antibodies for immunoblotting (IB) and Immunohistochemistry (IHC)

Antibody target	Host	Supplier	Dilution	Application
Vinculin	Mouse	Cell signalling	1:1000	IB
Id2	Mouse	D39E8<br>Cell Signalling	1:1000	IB
Aldh3a1	Goat	ARG65260<br>arigobio	1:10,000	IB
Lamin A/C	Rabbit	Santa Cruz Biotech (sc-2004)	1:500	IHC
CD31	Rat	Dianova (DIA310)	1:75	IHC
Endomucin	Rat	Santa Cruz Biotech (V.7C7; sc-65495)	1:1000	IHC

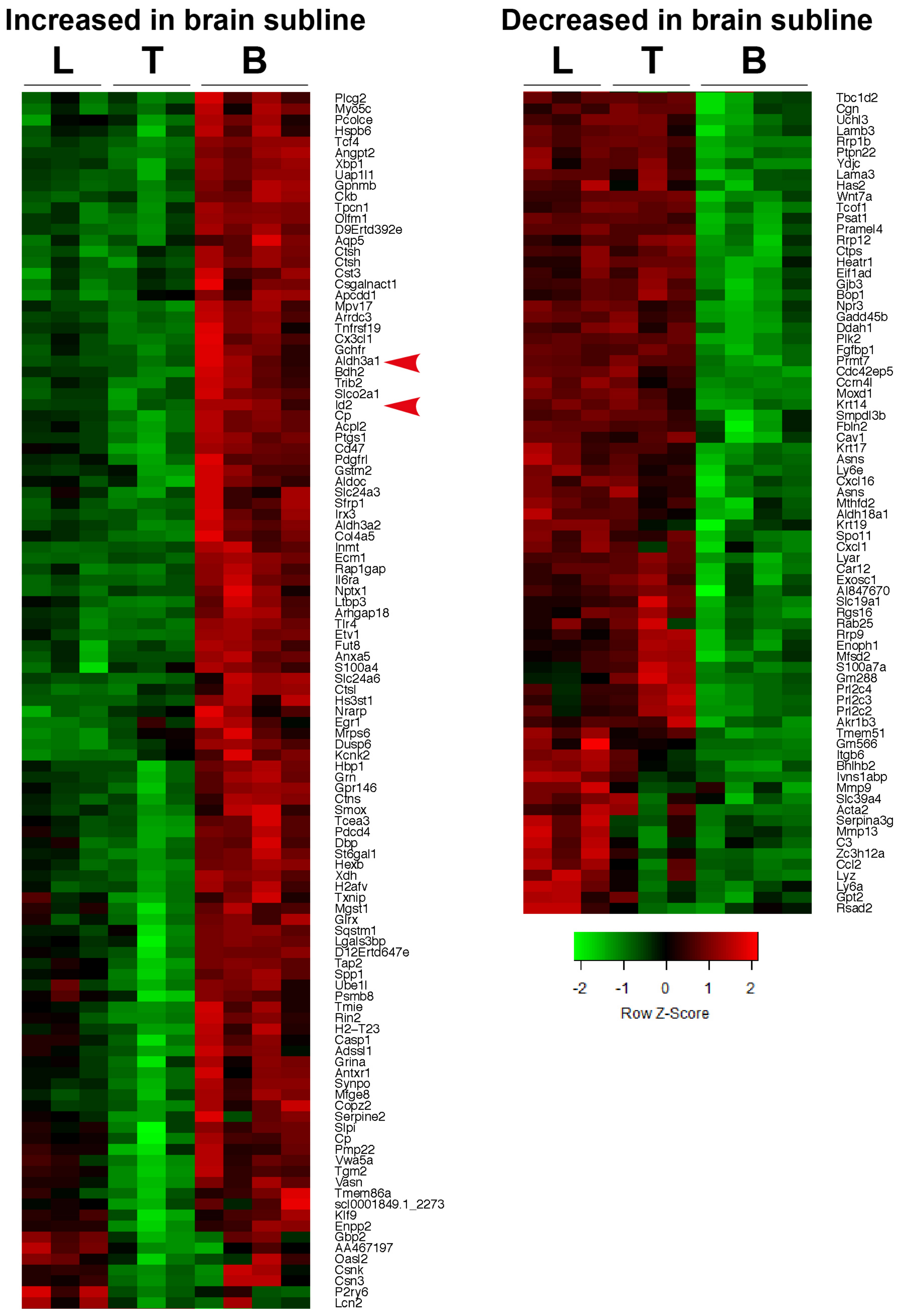

SFig. 1 Higher power image of Fig. 1c. Heat map (Pearson, ward.D2) of 186 genes (with official mouse gene symbol) differentially expressed between B and T, between B and L, or between L and T sublines with an absolute fold change ≥2.0, p<0.001. Sublines are in the same order as in Fig. 1a. Arrowheads indicate *Id2* and *Aldh3a1*.

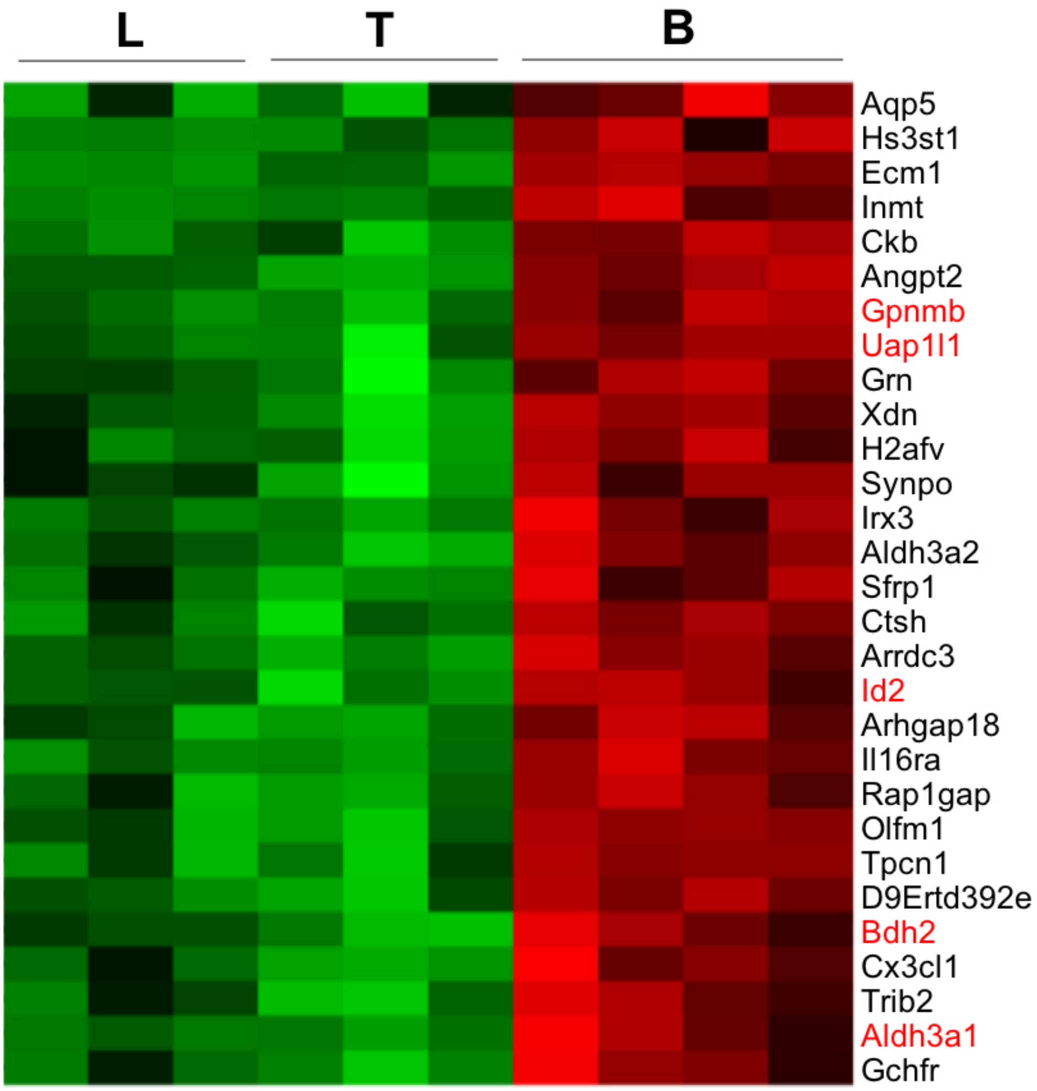

SFig. 2. Heat map of shortlisted genes. Heat map (Pearson, ward.D2) of 29 genes (with official mouse gene symbol) with upregulated expression (absolute fold change ≥2.0, p<0.001) in B versus T and B versus L but not between L and T. Genes further validated by RT-qPCR are shown in red.
